# Instrumental Texture Differentiation of Channel (*Ictalurus punctatus*) and Hybrid (Channel × Blue, *Ictalurus furcatus*) Catfish Fillets

**DOI:** 10.3390/foods11131875

**Published:** 2022-06-24

**Authors:** John M. Bland, Ryan Ardoin, Carissa H. Li, Peter J. Bechtel

**Affiliations:** Southern Regional Research Center, ARS, USDA, 1100 Allen Toussaint Blvd., New Orleans, LA 70124, USA; ryan.ardoin@usda.gov (R.A.); carissa.li@usda.gov (C.H.L.); peter.bechtel@usda.gov (P.J.B.)

**Keywords:** channel catfish, hybrid catfish, instrumental texture profile analysis, thickness covariance, cold-storage type

## Abstract

An analysis of instrumental texture differences between channel (*Ictalurus punctatus*) and hybrid (female channel × male blue, *I. furcatus*) catfish fillets is presented. Factors including cold-storage type (fresh, frozen, or individually quick frozen (IQF)) and gender were included in the comparisons. Texture was measured at eight positions per fillet by a texture profile analysis (TPA) method that provided seven texture attributes: firmness, toughness, cohesiveness, adhesiveness, chewiness, resilience, and springiness, plus the thickness of the fillets (238 total). All attributes except adhesiveness were found to be statistically different (*p* < 0.05) between channel and hybrid fillets, with channels having the highest value in each attribute. When cold-storage type was included in the analysis, channels still produced the highest attribute values, but the number of attributes differed with firmness, toughness, and chewiness most associated with the differences in the type of catfish, while the other attributes were affected by cold-storage type. Thickness was found to be a strong covariant to some of the texture attributes, especially toughness, but the determination of difference between channels and hybrids was not affected and TPA profiles provided high levels of differentiation between catfish types.

## 1. Introduction

Catfish fillet quality is dependent on flavor, odor, color, and texture attributes that are directly associated with their chemical and nutritional composition. Texture can be a very important parameter, as a sensory characteristic for consumers and for the mechanical properties needed by processors [1,2], and is influenced by several factors such as catfish age, size, growth rate, and species [3,4]. Consumers generally prefer a firm catfish fillet [5,6] because of the association of loss of firmness with the breakdown of muscle structure due to poor product quality.

Catfish (species of the order Siluriformes) represents the largest segment of U.S. aquaculture [7,8], where channel catfish (*Ictalurus punctatus*) has been the primary cultured species. However, as the industry was pressured to increase productivity since 2012, and higher-performing hybrid catfish lines (♀ channel catfish, I. punctatus × ♂ blue catfish, *I. furcatus*) were adopted that had a faster growth rate, a high tolerance to crowding and stress, and a better tolerance to low oxygen levels [9]. The crowding tolerance allowed hybrids to be stocked at higher levels, and with the implementation of new pond system technologies with increased capacity, such as intensive aeration and partitioned split-ponds, the productivity (yield) of catfish farms substantially increased from 4000 kg/ha in 2014 to 6000 kg/ha in 2019 [7]. Hybrid catfish has now surpassed channel catfish as the predominant U.S. aquaculture catfish type, comprising greater than 60% of catfish processed in 2020 [10].

In addition to the hybrid/channel genotype-environment interaction of crowding associated with stocking density, other stress factors that commonly affect cultured fish, especially in more intense culture practices, could cause differential side-effects and stress responses between channel and hybrid catfish, resulting in texture differences [11]. Stressors include changes in environmental temperature, dissolved oxygen levels, light intensity, water quality, handling, and transport [12].

Acute and chronic stress is correlated with increased metabolism, measured commonly as hyperglycemia, resulting in changes in growth rate, condition factors, or food conversion efficiency [13]. A 42% growth rate reduction was found from a 20% increase in metabolic rate for largemouth bass (*Micropterus salmoides*) [14]. High stocking density has been shown to induce a stress response in grass carp with reduction in weight and survival [15]. Increased metabolism can also reduce blood oxygen content, thus crowding stress could amplify low pond oxygen levels. Environmental and handling stress has also been shown to cause changes in the catfish muscle proteome with increases in structural and metabolic proteins but variable changes in texture [16]. A correlation between texture and muscle fiber size has also been demonstrated [17,18,19,20].

With increased aeration in ponds with hybrid catfish, there can also be an increased water flow rate, current, or mixing within the pond or the fish containment area [21,22]. This could raise the swimming activity for hybrids, causing changes in the muscle proteome structure and/or fat content of the resulting fillet, and thus textural changes.

With the advent of hybrid catfish, which are usually harvested as a single-batch, as compared to multiple-batch systems more common with channel catfish, the year-round harvest of hybrids can only be managed by stocking with different sizes/ages of catfish or extending the harvest through winter. This could affect the nutrient and chemical composition, such as the fatty acid or amino acid profile of the fillet, or protein and fat composition could be altered and affect texture. Kim and Lovell [23] found that channel catfish not fed during the winter lost 10% of their weight and significantly reduced fat content, condition factor, and muscle fiber size.

Catfish fillets are processed for cold storage as fresh fillets on ice or treated with polyphosphate prior to being individually quick frozen (IQF). Otherwise, fillets can be transferred to a freezer and frozen without polyphosphate treatment. A quick-freezing method, such as IQF, results in less cell damage from ice crystal growth and results in the retention of texture quality [24,25].

We have previously compared instrumental analysis of catfish fillets with sensory analysis [26], where predictive equations were developed for sensory attributes from various texture profile analysis (TPA) attributes. However, the study did not account for potential differences between channel and hybrid catfish. In the present study, we examined the instrumental textural differences between baked channel and hybrid catfish fillets in addition to effects of cold-storage type on TPA profiles by combining measurements from 94 previous fillets [26] with those from an additional 144 experimental units. The objectives of this research were to provide a thorough analysis of texture-related differences between channel and hybrid catfish fillets, the influence of cold-storage type (fresh, frozen, or IQF) on these physical differences, and the ability of TPA profiles to differentiate channel and hybrid catfish fillets.

## 2. Materials and Methods

### 2.1. Samples

Catfish fillet samples designated as frozen (25 channel and 25 hybrid) were obtained from catfish harvested from an experimental pond in Stoneville, MS. The catfish included a variety of families resulting from multiple spawns of both channel and hybrid (male blue x female channel) catfish. Hybrids were produced by strip spawning on two different days, while the channels were from pond spawning. Fish were reared as fry in separate family tanks for about 10 months and fed a fingerling diet (35% protein, Fishbelt Feeds Inc, Moorhead, MS, USA) to satiation once daily. They were then tagged with individually coded pit tags on the left fillet and stocked communally in an earthen pond. They were fed a commercial foodfish diet (32% Delta Western Research Center, Indianola, MS, USA) daily from April through October, then fed once a week until harvest the following January. With an average age of 592 ± 9 days, the fish were seized from the pond and held in a cement raceway overnight at 11–16 °C (52–61 °F). The fish, with an average weight of 771 ± 129 g (1.7 lb), were electrically stunned by a 40V electric pulse (Sylvesters, Inc., Louisville, MS, USA), gender determined, beheaded (Baader 166, Baader North America, Indianola, MS, USA), gutted by hand, filleted (Baader 184) and trimmed by hand. Both fillets from each fish were weighed and stored individually in a low-density polyethylene (LDPE) storage bag. All fillets were quickly placed in a −20 °C freezer overnight, before being transported on ice to the research facility and stored at −20 °C. Because of the increased control on processing, the stocking weight, whole weight at processing, headed gutted weight, percent carcass (skin, guts, and headed removed percent of whole weight), and age at processing were also recorded.

Individually quick frozen (IQF) fillets were obtained in two seasons, winter and summer. The winter-harvested IQF catfish fillets (30 channel and 30 hybrid) were obtained from catfish harvested from multiple commercial ponds (within 4 miles apart in Alabama) in January and transported to a commercial processing plant by truck (<15 miles). They had been fed a commercial diet (32% AL Catfish Feed mill, Uniontown, AL, USA). After netting the previous night, they were socked, loaded, and shipped within a 2-hr span on the morning of processing. Fish were weighed, and those from 600 to 900 g were used for the study. Fillets were processed, including polyphosphate treatment (vacuum tumble marination), and IQF in a mechanical blast freezer. Both fillets from each catfish were collected from the processing line and stored individually in a LDPE storage bag, transported on ice to the research facility, and stored at −20 °C. The left fillets were used for instrumental texture profile analysis.

Summer-harvested IQF fillets (19 channel and 19 hybrid) were from a June harvest in Mississippi and processed by a separate processing plant in a similar manner to the winter-harvested IQF fillets, but with an injection polyphosphate treatment.

Fresh (not-frozen) catfish fillets (30 channel and 30 hybrid) were obtained from the same batch of fillets as the winter IQF fillets but were removed from the processing line before phosphate and IQF treatment. Samples were transported on ice to the research facility, refrigerated, and used for instrumental texture profile analysis (TPA) within 3–4 days. A portion of this batch (an additional 15 channel and 15 hybrid) was frozen for TPA analysis in combination with the frozen samples from the experimental Stoneville, MS pond.

### 2.2. Texture Profile Analysis (TPA)

**Figure 1:**
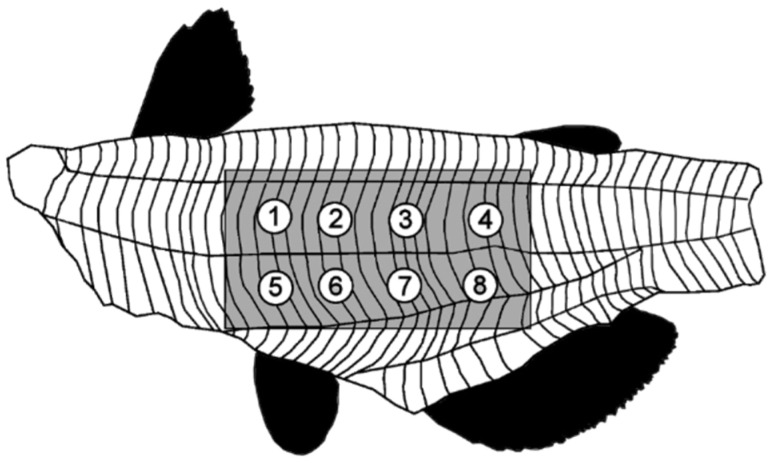
Positions 1−8 on fillet used for texture profile analysis. Shaded area was removed from the fillet before cooking [26].

Frozen and IQF fillets were thawed overnight in a refrigerator, weighed, and a middle rectangle, of dimensions 8.3 cm × 6.2 cm (head to tail × dorsal to ventral), was cut from the fillet (Figure 1, shaded area) to reduce variance in texture and cooking time. Each fillet section was weighed and a temperature probe (1/16” diameter, Pro-Series Needle Probe, cat #TX-1002X-NP), connected to a DOT alarm thermometer (ThermoWorks, American Fork, UT, USA), was inserted into the center of the fillet. The fillet was wrapped in aluminum foil that was perforated to allow steam to escape, placed on a cooking pan, and baked in a professional convection oven (Cyclone series, Bakers Pride Oven Co., Cheyenne, WY, USA) at 300 °F to an internal temperature of 165 °F (approx. 10 min). The fillet was removed and cooled to a surface temperature of approx. 86 °F (approx. 12 min) and placed on the texture analyzer (TA.XT plus, Texture Technologies, Hamilton, MA, USA).

**Figure 2:**
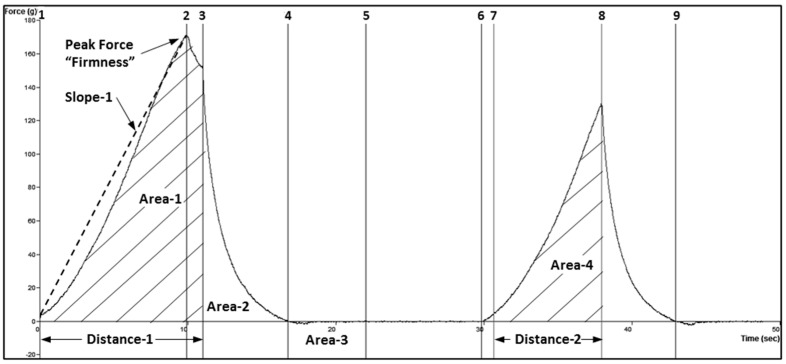
TPA force−time graph showing anchor points used to measure attributes. This was a non-representative sample that showed a separation between anchors 2 and 3.

Parameters for texture analysis were: texture profile analysis sequence of two compressions, 30 kg load cell, ½” diameter ball probe (TA-18), 5 g trigger force, 50% strain, 3mm/s pre-test speed, 1 mm/s test speed, 1 mm/s post-test speed, 5 s pause time between cycles. Eight positions (four on the dorsal side and four on the ventral side of the lateral line, 1.8 cm apart, as seen in Figure 1) on each fillet were tested. Force−time graphs (Figure 2) for each test point were analyzed with Exponent 32 software (Stable Micro Systems, Surrey, UK) using a self-written macro that determined the thickness of the fillet before and after compression, the maximum force of both compressions, the compression upstroke and downstroke energy, or work, as measured by area. Seven texture attributes and fillet thickness were calculated by the formulas provided in Table 1.

### 2.3. Proximate Analysis

Proximate analysis was performed on the frozen fillet samples. Moisture and ash content of catfish fillets were determined using AOAC (1990) methods #950.46, modified with lyophilization, and #923.03 [27], respectively. Chopped fillets were lyophilized in a VirTis Genesis 35EL freeze-dryer (SP Industries, Warminster, PA, USA), using a 7-day program and moisture content determined gravimetrically. Dried samples were placed in ceramic crucibles and incinerated in a muffle oven at 500 °C, followed by weighing to determine ash content. Nitrogen content was determined by pyrolysis with an FP628 nitrogen analyzer (Leco Co., St. Joseph, MI, USA). Protein content was calculated as 6.25 times the percent nitrogen. Total lipid content was determined gravimetrically by a modification of the Folch procedure [28] using a Dionex ASE 350 accelerated solvent extractor (Thermo Fisher Scientific, Waltham, MA, USA) where the lyophilized samples were transferred to 34 mL ASE cells and extracted with methylene chloride at 100 °C and 1500 psi into pre-weighed 60 mL vials. The solvent was removed in vacuo at 35 °C using a RapidVap Vacuum Evaporation System (Labconco Co., Kansas City, MO, USA). Moisture, ash, and lipid contents were determined in duplicate, and protein content was determined in triplicate for each replicate sample.

### 2.4. Statistical Analysis

For every fillet measured, an average value for thickness and each of the seven TPA attributes of interest (Table 1) was calculated from the eight compression positions and used for subsequent statistical analyses. Shapiro–Wilk tests were used to test normality of response variables. Due to deviations from normality when comparing overall instrumental texture differences between channel and hybrid fillets (without accounting for cold-storage type), a nonparametric Wilcoxon two-sample test was employed. When TPA data were sorted by cold-storage type (fresh, frozen, or IQF), the sub-sets became more normally distributed and two-sample *t*-tests were used to compare channel and hybrid fillets. An unbalanced analysis of variance (ANOVA) with Tukey’s HSD post-hoc test was used to identify differences in TPA attributes due to cold-storage type across all fillets (without separating channel and hybrid), as lack of balance does not present the same issues for single factor analysis as with factorial designs [29,30]. Canonical discriminant analysis was used to reduce the dimensionality of the response set and provided correlations of each TPA attribute with the overall variability between hybrid and channel texture profiles. Fisher linear discriminant analysis was used to create equations to predict group membership (channel or hybrid) based on TPA profiles. Pearson’s correlation coefficients were determined to evaluate linear relationships between fillet thickness and firmness, and for toughness and firmness between channel and hybrid fillets. The means of proximate analysis data and catfish production data were compared using ANOVA with Holm-Sidak post-hoc tests. Microsoft Excel (2019), SAS (Copyright© 2016 SAS Institute Inc., Cary, NC, USA), and SAS Enterprise Guide (Copyright© 2017 SAS Institute, Inc., Cary, NC, USA) were used for analyses. A significance level of α = 0.05 was used for all analyses.

## 3. Results

Channel and hybrid fillet samples were obtained from multiple sources and preserved using different cold-storage types prior to testing (fresh, frozen, or IQF). Eight positions on the fillet were tested using a ½ inch spherical probe to account for fillet location differences and differences in fillet thickness. The results of the texture profile analysis (TPA) produced seven instrumental texture metrics of interest, calculated as shown in Table 1. The firmness texture attribute, also termed as hardness in many TPA studies and the most prevalent texture attribute reported, was measured as the maximum force (g) of the first compression [31]. For the present discussion, the term firmness was chosen, as this descriptor may be more aligned with language used to describe sensorial texture of fish fillets [26]. For the same reason, toughness was used to describe the work required during the compression and has been considered a more useful measurement for correlation to sensory-determined firmness [32]. The other attributes measured were cohesiveness, springiness, chewiness, resilience, and adhesiveness. Additionally, the thickness of the fillets was measured from the first compression. In recent studies, hardness, cohesiveness, chewiness, resilience, and springiness have been considered the most relevant properties for instrumental fish texture analysis [31].

Comparing baked channel and hybrid catfish fillets across all three cold-storage types combined, six of the seven TPA attributes measured (firmness, toughness, cohesiveness, chewiness, resilience, and springiness) were significantly different between the two catfish types based on Wilcoxon two-sample tests (*p* < 0.05; Table 2). Values for channel fillets were greater than those for hybrid fillets for all attributes except adhesiveness. Firmness values have been reported elsewhere for hybrid and two strains of channel catfish with no clear patterns of differences [33]. In that study, baked hybrid fillets were significantly less firm than one strain of channel fillet, but not another. For fresh raw fillets, the hybrid was firmer than both strains of channels, and for frozen-thawed fillets, no significant difference was found. In another study, Johnson [34] reported TPA values for baked hybrid and channel catfish fillets, showing hybrids to be less firm than channels. Presently observed overall differences (Table 2) went beyond those related to peak force (i.e., firmness, toughness, and chewiness) and also indicated differences in textural properties (cohesiveness, resilience, and springiness) which have shown to behave independently of the firmness parameter in catfish products [35].

As fillet samples were processed and stored differently prior to analysis, TPA attributes were also compared between channels and hybrids for each cold-storage type using two-sample t-tests (Table 3). This analysis revealed how each cold-storage-type contributed to differences (or lack thereof) in texture-related physical properties between channel and hybrid fillets. For certain attributes, fresh fillets had the largest percent difference between channel and hybrid, with toughness, followed by chewiness, and firmness being more than 60% greater for channels. Among the fresh fillets, channels and hybrids significantly differed in five attributes, firmness, toughness, chewiness, resilience, and springiness. As opposed to the combined data (Table 2) and frozen fillet comparisons (Table 3), the significance of cohesiveness as a differentiating property was not found in fresh or IQF samples. Frozen fillet comparisons produced the same list of significant differences as the combined data, with six attributes being significantly different. This agreed with what had been reported by Johnson [34] on frozen fillets with a similar TPA method, even though samples in that study were refrigerated overnight after cooking. When alternative methods of Krammer shear force measurements were used, Park [36] and Bosworth et al. [33] showed channels to be statistically similar to hybrids in firmness, although slightly larger numerically.

However, among IQF processed fillets, only firmness, toughness, and chewiness were significantly different between channels and hybrids (Table 3). It is important to note that these three attributes all depend on the peak force measurement of the first compression. That is, they are all related to the primary TPA property hardness/firmness [31]. It had been reported that polyphosphate, and even the type of polyphosphate used in IQF fillets caused a reduction in firmness for channel catfish fillets [37]. IQF fillets in this study were only about 5% less firm than frozen fillets for both catfish types. Adhesiveness showed a larger percent decrease, especially for hybrids. IQF processing resulted in the fewest significant differences between channel and hybrid fillets among the three cold storage-types tested.

Combing data from both channel and hybrid fillets, we were able to examine the effects of cold-storage type alone (not biological type) on the textural properties themselves (Table 4). The three firmness-related attributes firmness, toughness, and chewiness significantly differed between hybrid and channel fillets both overall and within each cold-storage condition (Table 2 and Table 3, respectively). However, when combining data from both catfish types, cold-storage type did not account for the observed differences in any of these three texture properties, whereas cohesiveness, adhesiveness, resilience, and springiness did differ as a result of cold-storage treatment, based on ANOVA (Table 4). Therefore, the present analyses suggested that differences in firmness and its secondary texture properties toughness and chewiness are more related to catfish type (channel vs. hybrid) than cold-storage type, where channel catfish fillets were instrumentally firmer, tougher, and chewier.

**Figure 3:**
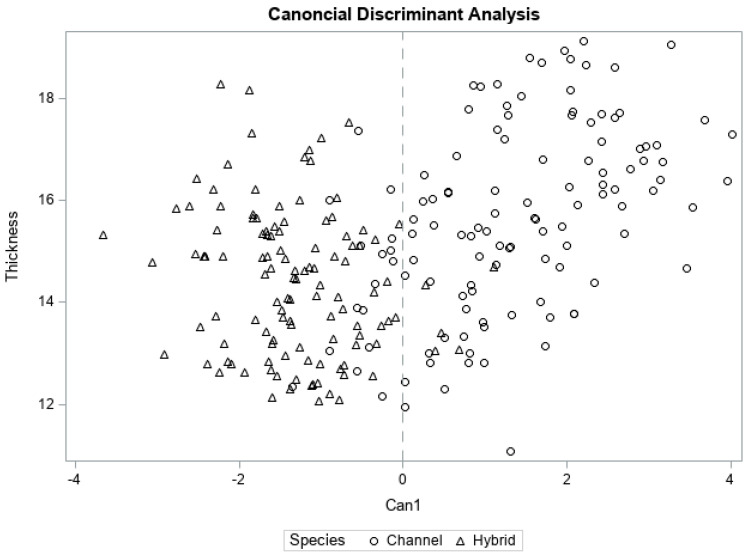
Group separation of channel and hybrid catfish fillets (*n* = 119) by TPA attributes. Because the variable catfish−type had two levels (channel and hybrid), the canonical discriminant analysis resulted in one canonical dimension (Can1) which accounts for 80% of the total variance explaining overall treatment differences.

Overall, firmness, chewiness, and toughness were the most discriminating TPA attributes among channel and hybrid fillets, having pooled within canonical correlations of 0.95, 0.84, and 0.71, respectively, with the first and only canonical dimension (Can1; Table 5). Reducing the overall dimensionality of the data to Can1 accounted for 80% of variability in the between-catfish type TPA dataset (Table 5, Figure 3). Considering the entire texture profiles (all seven attributes) as predictors, Fisher linear discriminant analysis (FLD) was able to correctly classify fillets as hybrid or channel with a high success rate (high hit rate; Table 6), although it should be noted these estimates may be overly optimistic when the same data used to create the FLD equation are also used to test the results [38]. The overall hit rate, or proportion of fillets correctly classified as channel or hybrid, was 0.921 overall. For fresh fillets, the model was able to successfully predict between channel and hybrid for 59 of 60 fillets (hit rate of 0.983). This high level of differentiation between fresh fillets may be related to the large corresponding differences in attribute magnitudes (Table 3). While human sensory data would be needed to determine whether observed statistical significance relates to differences in perception, and how such differences affect fillet acceptability, the current results have shown that TPA profiles can successfully differentiate baked channel and hybrid fillets within and across the three cold-storage types.

**Figure 4:**
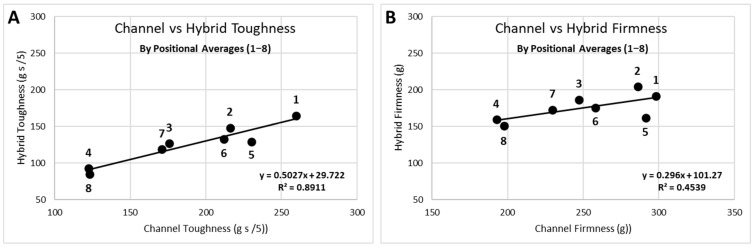
Data representation for parameters (channel vs hybrid) by positional averages for (**A**) toughness: Pearson’s correlation coefficient (r) = 0.94; and (**B**) firmness: correlation not significant.

**Figure 5:**
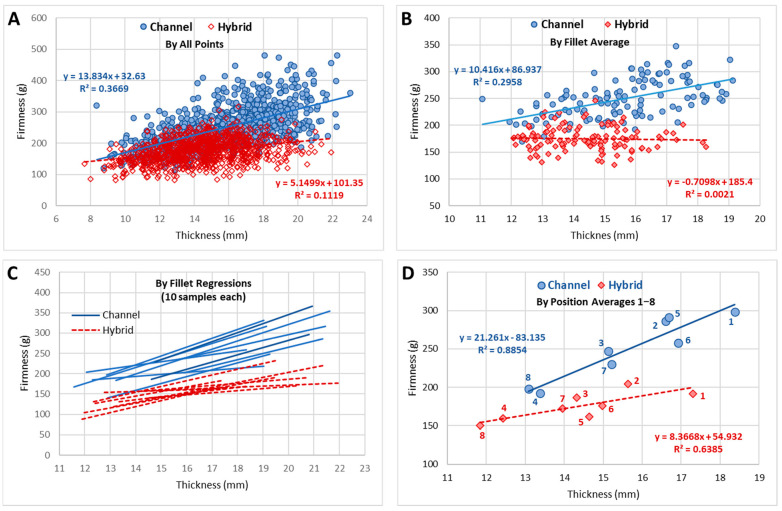
Data representation possibilities for attributes (thickness vs firmness). (**A**) All data points: Pearson’s correlation coefficient (r) = 0.61 for Channel, and r = 0.33 for Hybid; (**B**) Fillet averages: r = 0.54 for Channel, and no significant correlation for Hybrid; (**C**), Fillet 8-point regressions; (**D**) Position averages: r = 0.94 for Channel and r = 0.80 for Hybrid.

TPA data from the eight individual compression positions can be analyzed in several forms. Since fillets were not paired in this study, direct correlation of parameters, such as channel and hybrid, IQF and fresh, or cooked and raw, could only be meaningfully accomplished through the connection of the eight positions or the positional averages, as seen in Figure 4 for the toughness and firmness attributes. It was noteworthy that the correlation for toughness (Pearson’s correlation coefficient (r) = 0.94; Figure 4) was stronger than for firmness (correlation not significant at α = 0.05). The position-5 was known to be problematic, positioned at the edge of the nugget section of the fillet, and can be seen to have the maximum offset from the regression line. This correlated to position-5 having the largest standard deviation for all attributes. Indirect comparison of parameters could be obtained through correlation of two TPA attributes (e.g., thickness and firmness) for each of the two parameters. The attribute correlation can be accomplished with all data points (8 points per fillet × number of samples), the 8-point fillet averages, the fillet linear regression slopes, or the positional (1–8) averages (Figure 5).

The statistical comparisons of the texture attributes were found to be complicated with a covariance between fillet thickness and many of the TPA attributes, as seen in Figure 5 for the firmness attribute. If channels and hybrids were combined into a single model which fit the effects of thickness and of catfish type on firmness, and an interaction between the two, a large effect of thickness and of catfish type, plus a significant interaction were found, where the effect of thickness is greater for channel and less for hybrid. From the 95% confidence intervals of the model fit, a Johnson-Neyman (JN) point for the model could be calculated to determine the range of the significance of difference. However, because the regressions and CI converge below the thickness range, the JN point was undefined, indicating the whole range of data was significantly different, with channel being larger than hybrid. Alternatively, when the covariance of thickness and toughness was examined, the JN point was calculated to be 11.9 mm in thickness. Above this thickness, the predicted toughness for channel is higher than for hybrid. Since most of the data are above 11.9 mm thickness, the region of significance encompasses almost all the data. These low JN points assisted the previously discussed calculations of significance for the combined data and the cold-storage type data without considering the thickness covariance.

The channel and hybrid catfish used for the frozen fillet samples were obtained from special circumstances with both being stocked in the same experimental pond where they would be under identical environmental conditions and be harvested identically. Therefore, many variables associated with collecting channels and hybrids from different ponds would be eliminated and give rise to correlations with much less error. Moreover, the gender of the fish was determined for this sample type and information on the stocking, harvest, and processing weights and ages was available (Table 7). No significant differences were found between sample types except for Carcass percent, where channel and hybrid were significantly different and although male and female were not different, in total, the channel male or female significantly differed from the corresponding hybrid. Additionally, proximate data were obtained on the frozen samples (Table 8). The channel fillets had significantly less lipid than the hybrid, with the moisture and protein being slightly larger. When gender was included in the comparison, channel males had significantly more fillet moisture and protein and less lipid than comparable hybrid fillets. Channel and hybrid catfish normally have similar proximate levels with the average moisture, protein, and lipid content of channel catfish fillets being reported as 76.4%, 15.6%, and 6.9%, respectively [39], and hybrids with 77.8%, 16.7%, and 5.7%, respectively [40], both similar to the hybrid values in Table 8. It is unknown how channels and hybrids produced in the same pond for the frozen samples had such large differences in lipid content. Feeding competition or differences in fat loss during reduced feeding schedules may account for the differences. Higher fillet muscle lipid and moisture content has been associated with softer fish texture [41,42], or conversely, the reduced lipid levels in the channels of the frozen sample should be associated with an increase in firmness. This may be a factor in the observed 36% higher firmness value found for the channel frozen fillet compared to the hybrid (Table 3), but similar or larger firmness differences were found for the IQF and fresh samples that were not produced communally.

## 4. Conclusions

Channel and hybrid catfish fillets were well distinguished by the texture profile analysis method. Firmness, toughness, and chewiness texture attributes were most associated with the differences between channels and hybrids, while resilience, cohesiveness, adhesiveness, and springiness were associated with differences in the cold-storage types, fresh, frozen, and IQF. For all texture attributes, channels had higher values than hybrids, with fresh fillets having the largest percent difference. IQF fillets, containing polyphosphates, had the fewest number of texture attribute differences between the two catfish types. Thickness covariance was also detected for most of the texture attributes to varying degrees, but statistical analysis demonstrated that differences between channel and hybrid catfish fillets were significant for most of the sample thickness range encompassed by the data. Future sensory analysis should be conducted to investigate the effects of physical differences between channel and hybrid fillets on product acceptability.

## Figures and Tables

**Table 1:** Texture profile analysis attributes, with formula and description.

Attribute	Formula ^a^	Description
Thickness	2 × Distance 1	Fillet thickness—twice the 50% compression distance.
Firmness	Force at anchor 2	Maximum force of a 50% compression.
Toughness	Area 1/5	1st peak compression work, divided by 5.
Cohesiveness	Area 4/Area 1	2nd compression work relative to 1st compression work.
Springiness	Distance 2/Distance 1 × 100	Relative recovery from 1st compression.
Chewiness	Firmness × Cohesiveness × Springiness	Work required to chew sample to a state ready for swallowing.
Resilience	Area 2/Area 1 × 100	Decompression work relative to compression work.
Adhesiveness	Area 3	Negative work at end of decompression.

^a^ See Figure 2 for formula descriptors.

**Table 2:** Overall instrumental textural differences ^1^ between cooked Channel and Hybrid catfish fillets. ^2^ Values expressed as mean ± standard deviation (*n* = 119).

	Firmness (g)	Toughness (g × s)	Cohesiveness	Adhesiveness (g × s)	Chewiness (g)	Resilience (%)	Springiness (%)
Channel	**249.6 ± 35.3**	**187.6 ± 40.6**	**0.48 ± 0.03**	−1.1 ± 0.3	**85.8 ± 15.3**	**21.5 ± 1.8**	**71.0 ± 3.1**
Hybrid	**175.2 ± 21.9**	**124.6 ± 24.1**	**0.46 ± 0.03**	−1.1 ± 0.3	**56.6 ± 10.2**	**20.6 ± 1.8**	**68.4 ± 3.9**

^1^ Differences in TPA attributes were based on Wilcoxon two-sample test, at a significance level of α = 0.05. Values that differed between channel and hybrid, within columns, are in bold font. ^2^ TPA profiles were compared between cooked channel and hybrid catfish fillets, not accounting for raw fillet storage method (fresh, frozen, or IQF).

**Table 3:** Instrumental textural differences ^1^ between cooked channel and hybrid catfish fillets according to cold-storage type. Values expressed as means ± standard deviations.

	Fresh (*n* = 30)		Frozen (*n* = 40)		IQF (*n* = 49)	
	Channel	Hybrid	Channel	Hybrid	Channel	Hybrid
Firmness (g)	**274.6 ± 35.5**	**167.22 ± 18.7**	**248.3 ± 30.0**	**183.1 ± 24.2**	**235.3 ± 31.0**	**173.6 ± 20.0**
Toughness (g × s)	**216.3 ± 36.8**	**118.2 ± 19.6**	**178.8 ± 35.4**	**131.0 ± 32.1**	**177.2 ± 39.1**	**123.2 ± 17.3**
Cohesiveness	0.47 ± 0.03	0.46 ± 0.03	**0.47 ± 0.03**	**0.44 ± 0.02**	0.49 ± 0.02	0.48 ± 0.02
Adhesiveness (g × s)	−1.2 ± 0.3	−1.4 ± 0.3	−1.1 ± 0.3	−1.1 ± 0.3	−1.0 ± 0.2	−0.9 ± 0.3
Chewiness (g)	**93.4 ± 17.8**	**51.7 ± 8.4**	**84.7 ± 14.4**	**56.9 ± 10.3**	**81.9 ± 12.8**	**59.3 ± 10.2**
Resilience (%)	**22.5 ± 1.9**	**20.1 ± 1.8**	**21.3 ± 1.7**	**19.1 ± 1.1**	21.0 ± 1.7	21.5 ± 1.9
Springiness (%)	**71.1 ± 2.6**	**65.9 ± 2.3**	**71.5 ± 3.14**	**69.0 ± 4.2**	70.6 ± 3.1	69.4 ± 3.8

^1^ Differences in TPA attributes were based on *t*-tests, at significance level of α = 0.05. Values that differed between channel and hybrid, within each cold-storage type, are in bold font.

**Table 4:** Effects of cold-storage type on TPA attributes of catfish fillets. Values expressed as means ± standard deviations (*n* = 30 for fresh, *n* = 40 for frozen, *n* = 49 for IQF).

	Firmness (g)	Toughness (g × s)	Cohesiveness	Adhesiveness (g × s)	Chewiness (g)	Resilience (%)	Springiness (%)
Fresh	220.9 ± 61.0	167.2 ± 57.5	0.46 ± 0.03 ^b^	−1.3 ± 0.32 ^a^	72.5 ± 25.2	21.3 ± 2.2 ^a,b^	68.5 ± 3.6 ^b^
Frozen	215.7 ± 42.6	154.9 ± 41.3	0.46 ± 0.03 ^b^	−1.1 ± 0.28 ^b^	70.8 ± 18.7	20.6 ± 1.6 ^b^	70.3 ± 4.0 ^a^
IQF	204.5 ± 40.4	150.2 ± 40.5	0.48 ± 0.02 ^a^	−1.0 ± 0.25 ^c^	70.6 ± 16.2	21.3 ± 1.8 ^a^	70.0 ± 3.5 ^a^

^a–c^ Values in the same column followed by different superscripts were significantly different based on ANOVA with Tukey’s post-hoc test (α = 0.05).

**Table 5:** Pooled within canonical structure (*n* = 119) ^1^.

TPA Attribute	Can1
Firmness	0.95
Toughness	0.71
Cohesiveness	0.18
Adhesiveness	−0.01
Chewiness	0.84
Resilience	0.19
Springiness	0.28

^1^ Because the variable catfish−type had two levels (Channel and Hybrid), the canonical discriminant analysis resulted in one canonical dimension (Can1), which accounts for 80% of the total variance explaining overall treatment differences.

**Table 6:** Proportion of correct classifications (channel or hybrid; n-119) based on TPA.

	Hit Rate ^1^
Overall ^2^	0.912
Fresh	0.983
Frozen	0.875
IQF	0.888

^1^ Proportion of correct grouping between channel and hybrid fillets based on TPA profiles. ^2^ Not accounting for cold-storage type.

**Table 7:** Catfish production and processing data for frozen hybrid and channel fillets.

	Samples (No.)	Age (Days)	STWT (g)	WT (g)	HGWT (g)	Carcass (%)	Fillet (g)
total	98	592.3	90.2	771.2	507.7	65.4 ^b^	260.5
male	52	592.3	91.4	803.0	527.7	65.0 ^a,b^	268.3
female	46	592.2	88.8	735.2	485.0	66.0 ^b,c^	251.9
Channel	49	590.4	96.3	764.2	490.5	64.3 ^a^	256.4
C-male	26	590.5	99.6	793.8	504.4	63.7 ^a^	261.8
C-female	23	590.3	92.7	730.8	474.8	65.0 ^a,b^	250.3
Hybrid	49	594.2	84.0	778.1	524.8	66.6 ^c^	264.7
H-male	26	594.2	83.3	812.2	551.0	66.3 ^b,c^	275.0
H-female	23	594.2	84.9	739.6	495.3	67.0 ^c^	253.4

STWT = Stocking weight. WT =Whole weight at processing. HGWT = Headed gutted weight. Carcass = percent whole weight after removal of skin, gut, and head. Fillet = Combined fillet weight. ^a–c^ Values in the same column followed by different superscripts were significantly different based on ANOVA with Holm-Sidak post-hoc test (α = 0.05).

**Table 8:** Proximate compositions (wet weight basis) of raw frozen catfish fillets.

	Moisture	Protein	Lipid	Ash
Channel (*n* = 25)	76.1 (±1.6) ^b^	19.7 (±1.1) ^b^	3.7 (±1.2) ^a^	1.10 (±0.07) ^a^
C-male (*n* = 13)	76.6 (±1.5) ^b^	19.7 (±1.1) ^b^	3.3 (±1.1) ^a^	1.11 (±0.07) ^a^
C-female (*n* = 11)	75.5 (±1.6) ^b^	19.7 (±1.2) ^b^	4.3 (±1.1) ^a^	1.10 (±0.06) ^a^
Hybrid (*n* = 25)	74.6 (±1.4) ^a,b^	18.8 (±0.7) ^a,b^	5.8 (±1.3) ^b^	1.05 (±0.07) ^a^
H-male (*n* = 11)	74.9 (±1.1) ^a^	18.7 (±0.8) ^a^	5.6 (±1.0) ^b^	1.07 (±0.44) ^a^
H-female (*n* = 14)	74.4 (±1.7) ^a^	18.9 (±0.7) ^a,b^	6.0 (±1.6) ^b^	1.04 (±0.29) ^a^
Total frozen (*n* = 48)	75.3 (±1.7)	19.3 (±1.0)	4.8 (±1.6)	1.08 (±0.07)
T-male (*n* = 24)	75.8 (±1.5)	19.2 (±1.1)	4.4 (±1.5)	1.09 (±0.06)
T-female (*n* = 25)	74.9 (±1.7)	19.3 (±1.0)	5.2 (±1.6)	1.07 (±0.08)

^a,b^ Values in the same column followed by different superscripts were significantly different based on ANOVA with Holm-Sidak post-hoc test (α = 0.05).

## Data Availability

The data presented in this study are available wholly within the manuscript.

## References

[1] Hyldig G., Nielsen D. (2001). A review of sensory and instrumental methods used to evaluate the texture of fish muscle. J. Texture Stud..

[2] Kiessling A., Ruohonen K., Bjornevic M. (2006). Muscle fiber growth and quality in fish. Arch. Tierz. Dummerstorf.

[3] Love R.M. (1983). Texture and the fragility of fish muscle cells. Research at the Torry Research Station. J. Texture Stud..

[4] Barroso M., Careche M., Borderias A.J. (1998). Quality control of frozen fish using rheological techniques. Trends Food Sci. Technol..

[5] Rasmussen R.S. (2001). Quality of farmed salmonids with emphasis on proximate composition, yield and sensory characteristics. Aquacult. Res..

[6] Skjervold P.O., Røra A.M.B., Fjæra S.O., Vegusdal A., Vorre A., Einen O. (2001). Effects of pre-, in-, or post-rigor filleting of live chilled Atlantic salmon. Aquaculture.

[7] Engle C.R., Hanson T., Kumar G. (2021). Economic history of U.S. catfish farming: Lessons for growth and development of aquaculture. Aquac. Econ. Manag..

[8] Kumar G., Engle C., Hegde S., van Senten J. (2020). Economics of U.S. catfish farming practices: Profitability, economies of size, and liquidity. J. World Aquacult. Soc..

[9] Dunham R., Masser M. Production of Hybrid Catfish. SRAC Publication No. 190, June 2012 Revision, 1–7. https://aquaculture.ca.uky.edu/sites/aquaculture.ca.uky.edu/files/srac_0190_production_of_hybrid_catfish_0.pdf.

[10] Venugopalan A., Griffin M.J., Wise D.J., White D., Ford L., López-Porras A., Camus A.C., Hanson L.A. (2021). Virulence and immunogenicity of blue catfish alloherpesvirus in channel, blue and blue × channel hybrid catfish. J. Fish Dis..

[11] Ashley P.J. (2007). Fish welfare: Current issues in aquaculture. Appl. Anim. Behav. Sci..

[12] Dunham R.A., Brummett R.E., Ella M.O., Smitherman R.O. (1990). Genotype-environment interactions for growth of blue, channel and hybrid catfish in ponds and cages at varying densities. Aquaculture.

[13] Wendelaar Bonga S.E. (1997). The stress response in fish. Pysiol. Rev..

[14] Rice J.A. (1990). Bioenergetics modelling approaches to evaluation of stress in fish. Am. Fish. Soc. Symp..

[15] Adineh H., Naderi M., Nazer A., Yousefi M., Ahmadifar E. (2021). Interactive effects of stocking density and dietary supplementation with Nano selenium and garlic extract on growth, feed utilization, digestive enzymes, stress responses, and antioxidant capacity of grass carp, *Ctenopharyngodon Idella*. J. World Aquacult. Soc..

[16] Ciaramella M.A., Nair M.N., Suman S.P., Allen P.J., Schilling M.W. (2016). Differential abundance of muscle proteome in cultured channel catfish (*Ictalurus punctatus*) subjected to ante-mortem stressors and its impact on fillet quality. Comp. Biochem. Physiol. Part D.

[17] Hatae K., Yoshimatsu F., Matsumoto J.J. (1984). Discriminative characterization of different texture profiles of various cooked fish muscles. J. Food Sci..

[18] Hatae K., Yoshimatsu F., Matsumoto J.J. (1990). Role of muscle fibers in contributing firmness of cooked fish. J. Food Sci..

[19] Hurling R., Rodell J.B., Hunt H.D. (1996). Fiber diameter and fish texture. J. Texture Stud..

[20] Periago M.J., Ayala M.D., Lopez-Albors O., Abdel I., Martinez C., Garcia-Alcazar A., Ros G., Gil F. (2005). Muscle cellularity and flesh quality of wild and farmed sea bass *Dicentrarchus labrax* L.. Aquaculture.

[21] Hargreaves J.A. Pond Mixing. SRAC Publication No. 4602. 2003. pp. 1–6. https://srac.tamu.edu/fact-sheets/serve/168.

[22] Tucker C. Pond Aeration. SRAC Publication No. 3700. 2005. pp. 1–8. https://srac.tamu.edu/fact-sheets/serve/292.

[23] Kim M.K., Lovell R.T. (1995). Effect of overwinter feeding regimen on body weight, body composition and resistance to *Edwardsiella ictaluri* in channel catfish, *Ictarulus punctatus*. Aquaculture.

[24] Goswami T.K. Cryogenic fish freezing: Science, technology & economics. Proceedings of the 4th International Conference on Mechanical Engineering.

[25] Makri M., Melvin M., Hotos G., Doubi X. (2007). The biochemical and sensory properties of gilthead sea bream (*Sparus aurata*) frozen at different characteristic freezing times. J. Food Qual..

[26] Bland J.M., Bett-Garber K.L., Li C.H., Brashear S.S., Lea J.M., Bechtel P.J. (2018). Comparison of sensory and instrumental methods for the analysis of texture of cooked individually quick frozen and fresh-frozen catfish fillets. Food Sci. Nutr..

[27] AOAC (1990). Official Methods of Analysis of the AOAC.

[28] Folch J., Lees M., Sloane-Stanley G.H. (1957). A simple method for the isolation and purification of lipids from animal tissues. J. Biol. Chem..

[29] Milliken G.A., Johnson D.E. (2009). Analysis of Messy Data, Volume 1: Designed Experiments.

[30] Shaw R.G., Mitchell-Olds T. (1993). ANOVA for unbalanced data: An overview. Ecology.

[31] Bernardo Y.A.d., do Rosario D.K.A., Monteiro M.L.G., Mano S.B., Delgado I.F., Conte-Junior C.A. (2022). Texture profile analysis: How parameter settings affect the instrumental texture characteristics of fish fillets stored under refrigeration?. Food Anal. Methods.

[32] Texture Technologies Overview of Texture Profile Analysis. http://www.texturetechnologies.com/resources/texture-profile-analysis.

[33] Bosworth B.G., Wolters W.R., Silva J.L., Chamul R.S., Park S. (2004). Comparison of production, meat yield, and meat quality traits of NWAC103 line channel catfish, Norris line channel catfish, and female channel catfish x male blue catfish F1 hybrids. N. Am. J. Aquacult..

[34] Johnson A. (2021). Analyses of Texture and Sensory Traits, Carcass Traits, and Fillet Color in Different Genetic Types of Farmed Catfish: Genetic Approaches to Enhance Catfish Fillets. Master’s Thesis.

[35] Wiles J.L., Green B.W., Bryant R. (2004). Texture profile analysis and composition of minced catfish product. J. Texture Stud..

[36] Park S. (1998). Raw and Baked Aquacultured Catfish Quality Parameters. Master’s Thesis.

[37] Kin S., Schilling M.W., Smith B.S., Silva J.L., Jackson V., Kim T.J. (2010). Phosphate type affects the quality of injected catfish fillets. J. Food Sci..

[38] Truxillo C., Hamer R., Huber M., Rothenberg L., Tao J. (2008). Multivariate Statistical Methods: Practical Research Applications Course Notes.

[39] Nettleton J.A., Allen W.H., Klatt L.V., Ratnayake W.M.N., Ackman R.G. (1990). Nutrients and chemical residues in one-to two-pound Mississippi farm-raised channel catfish (*Ictalurus punctatus*). J. Food Sci..

[40] Haque M.M. (2018). Proximate Composition, Retained Water, and Bacterial Load for Two Sizes of Hybrid Catfish (*Ictalurus furcatus* × *Ictalurus punctatus*) Fillets at Different Process Steps. Master’s Thesis.

[41] Dunajski E. (1980). Texture of fish muscle. J. Texture Stud..

[42] Prasad Thakur D., Morioka K., Itoh Y., Obatake A. (2003). Lipid composition and deposition of cultured yellowtail *Seriola quinqueradiata* muscle at different anatomical locations in relation to meat texture. Fish. Sci..

